# Reducing Neuron Apoptosis in the Pontine Micturition Center by Nerve Root Transfer for Restoration of Micturition Function after Spinal Cord Injury

**DOI:** 10.1155/2020/5615097

**Published:** 2020-08-04

**Authors:** Ronghua Yu, Gang Yin, Jianguo Zhao, Huihao Chen, Depeng Meng, Jiaqiang Zhang, Yaofa Lin, Zheng Xie, Chunlin Hou, Haodong Lin

**Affiliations:** ^1^Department of Orthopedic Surgery, Tongren Hospital, Shanghai Jiaotong University School of Medicine, Shanghai 200336, China; ^2^Department of Orthopedic Surgery, Changzheng Hospital, Second Military Medical University, Shanghai 200003, China; ^3^Department of Orthopedic Surgery, Shanghai General Hospital, Shanghai Jiaotong University School of Medicine, Shanghai 200080, China; ^4^Department of Orthopedic Surgery, Taikang Xianlin Drum Tower Hospital, Medical College of Nanjing University, Nanjing, Jiangsu Province 210046, China

## Abstract

**Objective:**

The rate of neuronal apoptosis increases after spinal cord injury (SCI). Anastomosing the normal nerve roots above the SCI level to the injured sacral nerve roots can enhance the functional recovery of neurons. Therefore, we evaluated the effect of sacral nerve root transfer after SCI on pontine neuronal survival.

**Methods:**

Sprague–Dawley rats were randomly divided into three groups: Group A, reconstruction of afferent and efferent nerve pathways of the bladder after SCI; Group B, SCI only; and Group C, control group. We examined pontine neuronal morphology using hematoxylin and eosin (H&E) staining after SCI and nerve transfer. Bcl-2 and Bax protein expression changes in the pontine micturition center were quantified by immunohistochemistry. The number of apoptotic neurons was determined by TUNEL staining. We examined pontine neuronal apoptosis by transmission electron microscopy (TEM) at different time points.

**Results:**

H&E staining demonstrated that the number of neurons had increased in Group A, but more cells in Group B displayed nuclear pyknosis, with the disappearance of the nucleus. Compared with Group B, Group A had significantly higher Bcl-2 expression, significantly lower Bax expression, and a significantly higher Bcl-2/Bax ratio. The number of apoptotic neurons and neuron bodies in Group A was significantly lower than that in Group B, as indicated by TUNEL staining and TEM.

**Conclusions:**

These findings demonstrate that lumbosacral nerve transfer can reduce neuronal apoptosis in the pontine micturition center and enhance functional recovery of neurons. This result further suggests that lumbosacral nerve transfer can be used as a new approach for reconstructing bladder function after spinal cord injury.

## 1. Introduction

After SCI, atrophy appears in the corresponding functional areas of the brain as neurons gradually disappear and become fibrotic or is replaced by the surrounding functional area [1–3]. The recovery of nerve function below the level of spinal injury has always been a conundrum. Animal experiments and clinical cases have demonstrated that anastomosing the anterior and posterior nerve roots above the injury level to the anterior and posterior sacral nerve roots controlling the bladder can simultaneously reconstruct the afferent and efferent pathways of the bladder and improve bladder function [4–7]. However, the structural changes in the brain after reconstruction of the bladder afferent and efferent pathways remain unclear. Few studies focus on changes that occur in the brain after nerve transfer. Therefore, in this study, anastomosis of the dorsal and ventral roots of the nerves above the injury level and the dorsal and ventral sacral nerve roots controlling the bladder was used to reconstruct the rat bladder–spinal cord–cerebral nerve afferent and efferent pathways. Bcl-2 and Bax protein expression changes were examined in the pontine micturition center to explore the role of neuronal apoptosis at various times after spinal cord injury and nerve transfer. In this way, changes in functional areas of the brain after nerve transfer were examined.

## 2. Materials and Methods

### 2.1. Animal Models and Experimental Groups

Adult female Sprague Dawley rats (*N* = 90) weighing 240-260 grams were purchased from the Experimental Animal Center of the Second Military Medical University. The rats were randomly divided into three groups, with 30 rats in each of the following groups: Group A, reconstruction of afferent and efferent nerve pathways of the bladder performed after SCI; Group B, SCI only; and Group C, control group.

**Figure 1:**
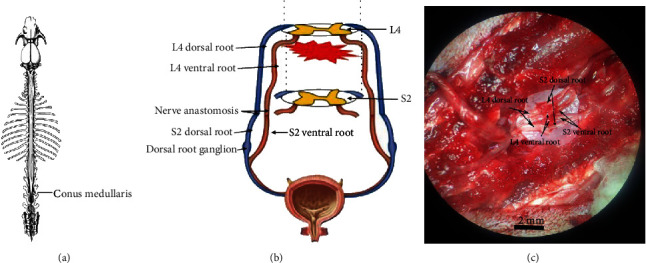
Mechanism of spinal cord injury and reconstruction of neural pathways. (a) Schematic diagram of the rat spine: the conus medullaris is at the level of L4. (b) Schematic diagram of the bladder function restoration surgery: the spinal cord and spinal nerves below L4 were cut, leading to blockade of afferent and efferent bladder–cerebral nerve pathways. The dorsal and ventral roots of bilateral S2 were anastomosed with the dorsal and ventral roots of bilateral L4. (c) Intraoperative image of the bladder function restoration surgery. Scale bar: 2 mm.

Rats were anesthetized with an intraperitoneal injection of 1% sodium pentobarbital at 40 mg/kg. The conus medullaris was exposed at the level of L4 (Figure 1(a)), and the spinal cord was completely cut below L4 with surgical scissors. In Group A, the bilateral L4 nerve roots were isolated from the endorhachis and cut at the intervertebral foramen. The bilateral S2 nerve roots were dissected and cut. 12-0 microsutures were used for tension-free suturing of the dorsal and ventral roots of bilateral S2 and L4 (Figures 1(b)–1(c)). In Group B, the severed spinal cord was not treated. In Group C, the spinal cord and spinal nerves below L4 were exposed but not lesioned. Following surgery, the rats were intraperitoneally injected with gentamicin (0.5 mg/kg) once daily for 3 days. Abdominal massage was performed twice daily (in the morning and evening) to help expel residual urine. The rats were sacrificed by intraperitoneal injection of 1% sodium pentobarbital solution at 1 day, 1 week, and 1, 3, or 6 months after surgery (6 rats in each group at each time point), and the dorsolateral pontine tegmentum was dissected.

### 2.2. Morphological Analysis

The dorsolateral pontine tegmentum and bladder tissues were postfixed with 4% paraformaldehyde, washed with normal saline, and dehydrated with increasing concentrations of ethanol. Longitudinal sections 5 *μ*m thick were sliced and retained for routine hematoxylin and eosin (H&E) staining. Ten visual fields were randomly selected, and structural changes in the dorsolateral pontine tegmentum were observed under a light microscope.

### 2.3. Electrophysiological Studies of the Bladder

Three months after surgery, the rats in each group were anesthetized with 1% pentobarbital, the bladder was emptied, and a 20 G intravenous indwelling blunt needle was inserted into the external urethral orifice. The intravenous indwelling needle was connected with a microinjection pump and pressure transducer, respectively, via a three-way tube, and the pressure transducer was connected to a physiological signal device (RM6240C Chengdu Instrument Factory). Intravesical instillation of saline at 37°C at a speed of 9 ml/h was performed to measure the pressure-volume curve. The S1 and S2 nerve roots of the rats were then dissected. The stimulus electrodes were placed at the distal end of the anastomosis of the dorsal roots of S2 and the posterior roots of the normal S1 nerve roots. Changes in bladder internal pressure were recorded. The stimulus intensity was 3 mA, the pulse width was 0.3 ms, the stimulus frequency was 20 Hz, and the duration was 5 s.

### 2.4. Immunohistochemistry for Bcl-2 and Bax Proteins

Tissue samples from the dorsolateral pontine tegmentum underwent routine deparaffinization, hydration, antigen recovery, and washing in phosphate-buffered saline (PBS). Normal goat serum blocking solution was added followed by the addition of Bcl-2 or Bax mouse monoclonal antibodies (anti-Bcl-2 antibody, ab194583, Abcam; anti-Bax antibody, ab32503, Abcam). After overnight incubation at 4°C, the sections were rewarmed at 37°C for 45 min. The sections were then washed in PBS three times for 5 min each. A secondary horseradish peroxidase–labeled goat anti-mouse antibody was added dropwise and incubated with the tissue at 37°C for 30 min. 3,3′-diaminobenzidine development was conducted for 5 min. After washing in PBS for 5 min, the tissue was counterstained with hematoxylin for 2 min and differentiated with hydrochloric acid and ethanol. The tissue was washed in PBS for 10 min, dehydrated, and cleared before being cover-slipped. The sections were incubated at 180°C in sodium citrate solution (H 3300, pH 6.0) for 15 min. The slides were allowed to cool down for 20 min at room temperature and were washed two times with distilled water. Five visual fields were randomly selected from each section under an inverted fluorescence microscope. ImageJ software (National Institutes of Health, Bethesda, MD) was used for analysis. Immunopositive cells were considered those with brown particles present in the cytoplasm. The proportion of the number of immunopositive cells (brown cytoplasm) to the total number of cells in a field was obtained, and a mean value was calculated for each section.

### 2.5. Apoptotic Cell Count

Samples from dorsolateral pontine tegmentum were deparaffinized and terminal deoxynucleotidyl transferase dUTP nick end labeling (TUNEL) staining was conducted. The sections were deparaffinized and rehydrated, rinsed in 0.1 M PBS for 10 min, and then reacted with proteinase K (20 *μ*g/mL) for 30 min at 37°C. After the sections were washed in PBS, they were incubated with an equilibration buffer (200 mM potassium cacodylate, 25 mM Tris-HCl, 0.2 mM DTT, and 0.2 mg/mL goat serum albumin) for 30 min at room temperature. The specimens were then incubated in labeling reaction mixture (containing TdT and dUTP) for 1 h at room temperature in the dark. Next, the sections were incubated with 2 x saline citrate (300 mM NaCl and 30 mM sodium citrate) for 10 min at 25°C, washed in PBS, and reacted with DAPI for 5 min at room temperature in the dark. A fluorescence microscope (Olympus) was used to acquire images. For the positive control, DNase I was added and incubated for 10 min at 25°C. dUTP was used for the negative control. Samples were observed under an inverted fluorescence microscope, and five visual fields were randomly selected from each section to determine the number of apoptotic cells.

### 2.6. Transmission Electron Microscopy (TEM)

Samples from dorsolateral pontine tegmentum measuring less than 1 mm^3^ in volume were cut, fixed with 2.5% glutaraldehyde, dehydrated with increasing concentrations of ethanol, embedded in paraffin, sectioned to 50–60 nm with an ultrathin microtome, stained with 3% uranyl acetate–lead citrate, and observed by TEM.

### 2.7. Statistical Analysis

Statistical analysis was performed using the SPSS 19.0 software (SPSS). Comparisons between groups were analyzed by two-way analysis of variance. *P* < 0.05 was considered statistically significant. Data were expressed as mean ± standard deviation.

## 3. Results

A total of three rats in Group A died at 1, 3, and 5 months after surgery, and a total of three rats in Group B died during the first week and at 3 and 4 months after surgery. The causes of death were thought to be wound infection or renal failure. All rats in Group C survived.

### 3.1. Bladder Function Recovery

**Figure 2:**
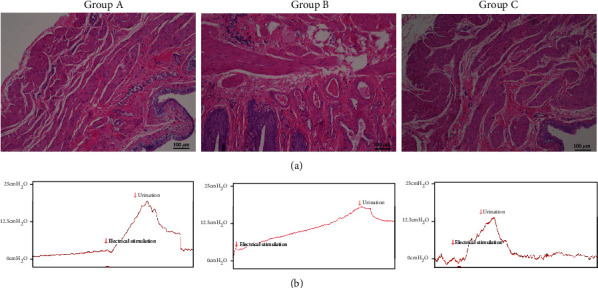
Morphological changes of the bladder and urodynamics at 3 months after surgery. (a) The bladders of the three study groups were histologically analyzed by H&E staining. The bladder mucosa from rats in Group A appeared smooth, and the muscle layer showed slight thickening. In rats from Group B, the bladder mucosa was rough and the mucosal cells appeared disordered. The muscle layer exhibited thickening and hypertrophy with fibrous tissue proliferation and inflammatory cell infiltration. In rats from Group C, the bladder mucosa was smooth and the muscle layer was thin, and the smooth muscle cells were small and arranged regularly. Scale bar: 100 *μ*m. (b) Electrophysiological features of the three study groups. The electrophysiological results for rats in Groups A and C at 3 months after surgery showed that after electrical stimulation, the intravesical pressure fluctuated drastically. The intravesical pressure did not fluctuate in Group B.

The bladder mucosa from rats in Group A (spinal cord lesion plus nerve root anastomosis) appeared smooth and the mucosal cell arrangement was regular. The muscle layer was slightly thickened, and smooth muscle cells were slightly hypertrophic and arranged regularly. In rats from Group B (spinal cord lesion alone), the bladder mucosa was rough, and the mucosal cells appeared disordered. The muscle layer was thickened and hypertrophic with fibrous tissue proliferation and inflammatory cell infiltration, and smooth muscle cells showed a disordered arrangement. In rats from Group C (control), the bladder mucosa was smooth and the mucosal cells were organized in a regular pattern. The muscle layer was thin, and the smooth muscle cells were small and arranged regularly (Figure 2(a)).

The electrophysiological results from rats in Groups A and C at 3 months after surgery showed that after electrical stimulation, the intravesicular pressure fluctuated drastically and urine was discharged. The intravesical pressure did not fluctuate in Group B (Figure 2(b)).

### 3.2. Morphological Analysis

**Figure 3:**
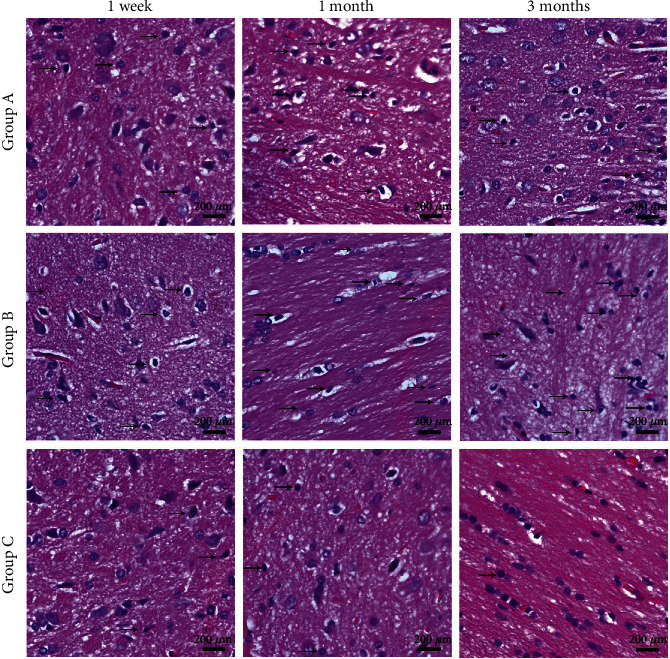
Pontine neurons in the three study groups, analyzed by H&E staining. At 1 week after surgery, the extracellular space around the neurons was slightly increased in Groups A and B, compared with that in Group C, and at 1 month after surgery, some neurons showed nuclear pyknosis in Groups A and B. Three months after surgery, accumulation of interstitial fibrous tissue was evident. Scale bar: 200 *μ*m.

H&E staining showed that the pontine neurons in rats from Group C were dense and arranged neatly, with clear nucleoli. One week after surgery, the extracellular space around the neurons was slightly increased in Groups A and B compared with that in Group C, and one month after surgery, some neurons showed nuclear pyknosis in Groups A and B. There was no significant difference in tissue morphology between Groups A and B when examined on the first week, and first month after surgery. Three months after surgery, an accumulation of interstitial fibrous tissue was evident. The number of neurons increased in Group A, but increasingly more cells in Group B displayed nuclear pyknosis, with the disappearance of the nucleus (Figure 3). This suggests that the apoptosis rate of neurons after nerve root transfer (Group A) was lower than that without nerve root transfer (Group B) at three and six months after surgery.

### 3.3. Immunohistochemistry for Bcl-2 and Bax Protein Expression

**Figure 4:**
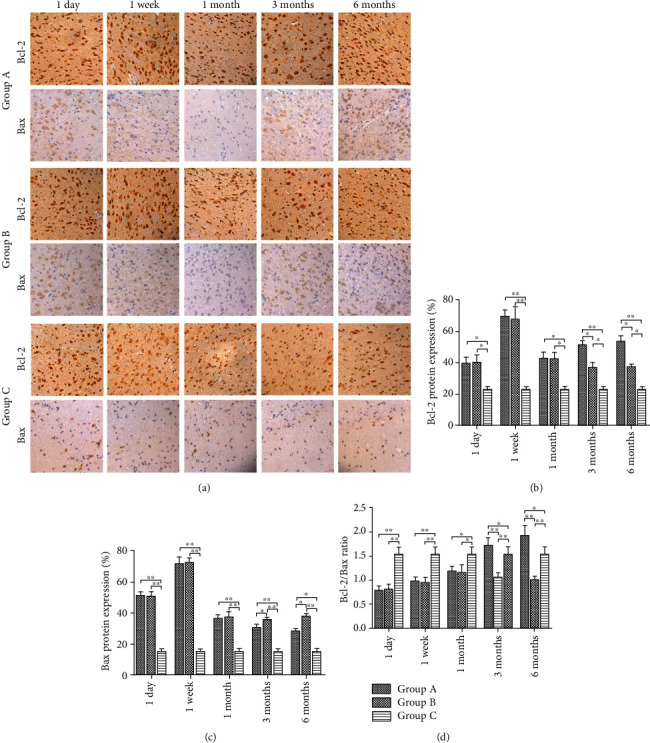
Bcl-2 and Bax protein expression in pontine neurons and the number of apoptotic neurons in the three study groups at each time point. (a) Bcl-2 and Bax protein expression in the three study groups under a fluorescence microscope (x200). (b) Fluorescence intensities of Bcl-2 protein expression in (a) were quantified by gray value analysis. Data represent the means ± SD. Two-way analysis of variance, ^∗^*P* < 0.05, ^∗∗^*P* < 0.01. (c) Fluorescence intensities of Bax protein expression in (a) were quantified by gray value analysis. Data represent the means ± SD. Two-way analysis of variance, ^∗^*P* < 0.05, ^∗∗^*P* < 0.01. (d) The Bcl-2/Bax ratios were measured by gray value analysis. Data represent the means ± SD. Two-way analysis of variance, ^∗^*P* < 0.05, ^∗∗^*P* < 0.01.

Neuronal Bcl-2 expression increased gradually in Group A and reached the highest level one week after surgery. The expression of Bcl-2 increased again gradually one month after surgery. In contrast, the expression of Bcl-2 in Group B decreased gradually over time. The intensity of Bcl-2 expression in the dorsolateral pontine tegmentum in Group A was significantly higher than Group B at 3 and 6 months after surgery (*P* < 0.05). The expression of Bcl-2 in Group C was minimally changed or unchanged at each time point. Statistically significant differences in Bcl-2 expression were observed between Group C and the other groups at each time point (*P* < 0.05) (Figures 4(a)–4(b)).

The mean expression of Bax gradually increased in Group A, reaching the highest level one week after injury, followed by a gradual decrease in expression over time. Bax expression gradually increased again one month after injury. Compared with Group B, the intensity of Bax expression in the dorsolateral pontine tegmentum in Group A was significantly lower at 3 and 6 months after surgery (*P* < 0.05). Bax expression in Group C was minimally changed or unchanged at each time point. Statistically significant differences in Bax expression were observed between Group C and the other groups at each time point (*P* < 0.01) (Figures 4(a) and 4(c)).

The mean ratio of Bcl-2/Bax in Group A gradually increased. The mean ratio of Bcl-2/Bax in Group B, however, increased gradually before one month and then gradually decreased. The Bcl-2/Bax ratio in Group C was minimally changed or unchanged at each time point. Statistically significant differences in the Bcl-2/Bax ratio were observed between Group C and the other groups at each time point (*P* < 0.05). Compared with Group B, the ratio of Bcl-2/Bax expression in Group A was significantly higher at three and six months after surgery (*P* < 0.01) (Figures 4(a) and 4(d)).

### 3.4. Number of Apoptotic Neurons

**Figure 5:**
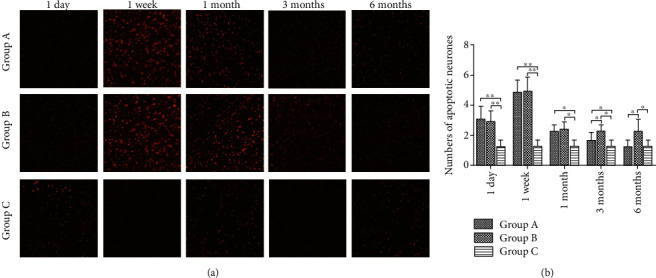
Number of apoptotic neurons, analyzed by TUNEL staining. (a) Number of apoptotic neurons in the three study groups at each time point, visualized by TUNEL staining (x100). (b) The number of apoptotic neurons was measured by gray value analysis. Data represent the means ± SD. Two-way analysis of variance, ^∗^*P* < 0.05, ^∗∗^*P* < 0.01.

The number of apoptotic neurons in Group A peaked at one week and then decreased gradually. The number of apoptotic neurons in Group B also peaked at one week and then decreased, but there was no significant change in the number of apoptotic neurons. Compared with Group B, the mean number of apoptotic neurons in Group A was significantly lower at three and six months after surgery (*P* < 0.05). The number of apoptotic neurons in Group C was minimally changed or unchanged at each time point. Statistically significant differences in the number of apoptotic neurons were observed between Group C and the other groups at one day and one week as well as one and three months after surgery (*P* < 0.05) (Figures 5(a)–5(b)).

### 3.5. TEM Analysis

**Figure 6:**
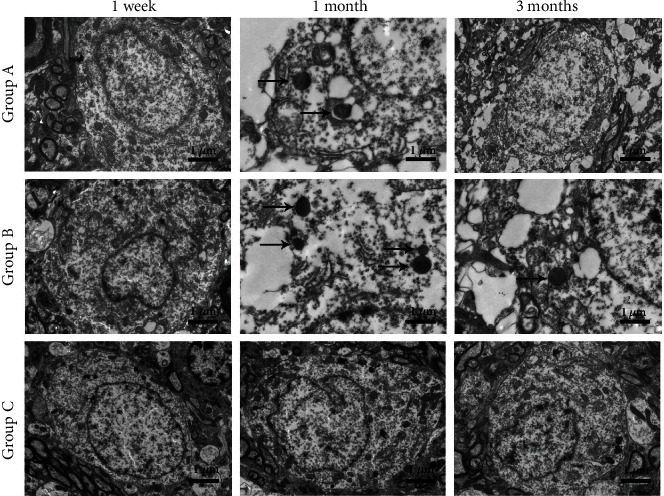
Pontine neurons of the three treatment groups, visualized by TEM. TEM analysis showed nuclear pyknosis and mitochondrial vacuolization in Groups A and B at 1 month after surgery, as well as pyknotic margination of chromatin, intercellular space enlargement, and apoptotic bodies. The number of apoptotic cells had increased in Group B. The arrows indicate apoptotic bodies. Scale bar: 1 *μ*m.

TEM showed that there were no significant changes in neurons in the three groups at 1 week after surgery. However, nuclear pyknosis and mitochondrial vacuolization were observed in Groups A and B at 1 month after surgery, along with pyknotic margination of chromatin, intercellular space enlargement, and apoptotic bodies. The number of apoptotic cells increased in Group B at 1 month after surgery. At 3 months after surgery, the neurons in Group A appeared plump, with an increase in organelle size and substantially more mitochondria compared to earlier time points (Figure 6). This suggests that the neurons in Group A were gradually recovering.

## 4. Discussion

Recent studies have shown that damage to the mammalian nervous system could cause changes in the functional plasticity of the brain [8, 9]. A study found that changes in the brain sensory cortex after central nervous system injury could lead to functional remodeling of the cerebral motor cortex [10]. Many subsequent studies confirmed that plasticity changes occurred in the brain after SCI [11–14]. However, functional remodeling of the brain after SCI did not always occur. Some corresponding brain functional zones underwent apoptosis, necrosis, degeneration, invasion by surrounding functional areas, or fibrosis after SCI [15, 16]. Experiments determined that the occurrence of brain degeneration or neuronal apoptosis was associated with the degree of SCI. Severe SCI caused apoptosis of hippocampal neurons in rats, whereas mild SCI did not cause obvious neuronal damage [17].

Thus, researchers started to study whether functional remodeling after SCI was also related to the degree of SCI. A study indicated that cerebral motor cortex activity in patients with SCI after remodeling was closely linked to the undamaged brain sensory cortex, suggesting an important role for negative sensory feedback in brain remodeling [18]. To confirm this, Wang et al. [19] subsequently performed a similar study in patients with complete SCI. After observing patients with complete SCI for 1 year, they found that negative sensory feedback was not extensively activated and no change in functional remodeling was detected in the motor cortex. In the case of complete SCI, the brain cortex is not extensively activated because the brain receives no afferent nerve stimulation. By contrast, for incomplete SCI, nerve conduction pathways are repaired and reconstructed through residual axons and nerves. Continuous nerve conduction pathways may be a necessary condition for brain remodeling. This suggests that reconstruction of the nerve pathway is beneficial for functional recovery of the corresponding brain regions. In cases of bladder dysfunction after SCI, early recovery of the nerve pathway is beneficial for preventing neuronal apoptosis and atrophy in related brain functional areas.

After SCI, restoration of function of the injured spinal cord is difficult. Nerve transfer is one of the most common methods used for the restoration of function of the injured spinal cord. After nerve transfer, the function of the corresponding distal nerve is partially restored, but the changes that occur in functional areas of the brain have not been studied. Lin et al. [4–7] reported that reconstructing the conduction pathway from the spinal cord to the brain through nerve repair or transplantation could promote brain remodeling. However, there are no studies on the changes in neurons in the brain. The function of neurons in the brain is determined based on the degree of recovery of function of the injured spinal cord [20]. Therefore, our research focused on the changes in neurons in the brain through nerve transfer after SCI. For rats in Group B (SCI only), light and electron microscopy results confirmed that neuronal apoptosis and degeneration occurred in the pontine micturition center. In Group A, cell regeneration and functional remodeling was evident in the pontine center at 3 and 6 months after surgery. We speculate that the reconstruction of neural pathways, especially the reconstruction of the bladder-to-cerebral afferent pathway, plays a critical role. In Group B, complete spinal cord injury led to afferent nerve block and no activation of the periaqueductal gray (PAG). Therefore, there was no functional remodeling in the pontine micturition center. However, in Group A, the afferent pathway of the bladder was reconstructed by anastomosing the anterior and posterior roots of S2 and L4, and the PAG could be extensively reactivated. The connection of the PAG with the cerebral cortex and Barrington's nucleus enabled the pontine micturition center to receive nerve stimulation again, which may be an important mechanism underlying its functional remodeling.

Apoptosis is a mechanism of cell self-death, and neurons tend to undergo apoptosis after injury or loss of negative feedback [21]. Bcl-2 and Bax are important proteins involved in cell apoptosis. Therefore, we studied the changes in Bcl-2 and Bax in neurons to analyze apoptosis. The expression of Bcl-2 and Bax increased the day after surgery, peaked at the first week after surgery. However, in Group A, Bcl-2 expression was elevated again at 3 and 6 months after surgery, whereas Bax expression continued to decrease, leading to a continuous increase in the Bcl-2/Bax ratio. In Group B, by contrast, Bcl-2 continued to decline, while Bax remained unchanged, resulting in a continuous decrease in the Bcl-2/Bax ratio. Because the Bcl-2/Bax ratio determines whether cells are apoptotic, this difference may be a mechanism underlying the repair and functional remodeling of nerve tissue observed in Group A and the neuronal apoptosis and degeneration observed in Group B. The mean number of apoptotic neurons in Group A at 3 and 6 months after surgery was significantly lower than that in Group B, consistent with the aforementioned results. Previous results showed that Bcl-2 inhibited cell apoptosis and Bax promoted cell apoptosis [22, 23]; further, the higher the ratio of Bcl-2/Bax, the stronger was the inhibition of cell apoptosis. Our study showed that the Bcl-2 level had increased and the Bax level had decreased in neurons, and the Bcl-2/Bax ratio had increased. These changes are indicative of inhibition of neuron apoptosis. This was confirmed by TUNEL staining and TEM, which showed that neuron apoptosis was decreased after nerve transfer.

## 5. Conclusion

Our findings demonstrate that nerve transfer can reduce apoptosis in corresponding neurons in the brain after SCI via changes in Bcl-2 and Bax expression. This result indicates that lumbosacral nerve transfer has potential as a new approach for restoring bladder function after SCI.

## Data Availability

The data used to support the findings of this study are available from the corresponding author upon request.
